# Functional Identification of APIP as Human mtnB, a Key Enzyme in the Methionine Salvage Pathway

**DOI:** 10.1371/journal.pone.0052877

**Published:** 2012-12-28

**Authors:** Camille Mary, Paula Duek, Lisa Salleron, Petra Tienz, Dirk Bumann, Amos Bairoch, Lydie Lane

**Affiliations:** 1 CALIPHO group, SIB-Swiss Institute of Bioinformatics, University of Geneva, Geneva, Switzerland; 2 Department of Human Protein Sciences, University of Geneva, Geneva, Switzerland; 3 Biologie of infection, Biozentrum, University of Basel, Basel, Switzerland; University of Turin, Italy

## Abstract

The methionine salvage pathway is widely distributed among some eubacteria, yeast, plants and animals and recycles the sulfur-containing metabolite 5-methylthioadenosine (MTA) to methionine. In eukaryotic cells, the methionine salvage pathway takes place in the cytosol and usually involves six enzymatic activities: MTA phosphorylase (MTAP, EC 2.4.2.28), 5′-methylthioribose-1-phosphate isomerase (mtnA, EC 5.3.1.23), 5′-methylthioribulose-1-phosphate dehydratase (mtnB, EC: 4.2.1.109), 2,3-dioxomethiopentane-1-phosphate enolase/phosphatase (mtnC, EC 3.1.3.77), aci-reductone dioxygenase (mtnD, EC 1.13.11.54) and 4-methylthio-2-oxo-butanoate (MTOB) transaminase (EC 2.6.1.-). The aim of this study was to complete the available information on the methionine salvage pathway in human by identifying the enzyme responsible for the dehydratase step. Using a bioinformatics approach, we propose that a protein called APIP could perform this role. The involvement of this protein in the methionine salvage pathway was investigated directly in HeLa cells by transient and stable short hairpin RNA interference. We show that APIP depletion specifically impaired the capacity of cells to grow in media where methionine is replaced by MTA. Using a *Shigella* mutant auxotroph for methionine, we confirm that the knockdown of APIP specifically affects the recycling of methionine. We also show that mutation of three potential phosphorylation sites does not affect APIP activity whereas mutation of the potential zinc binding site completely abrogates it. Finally, we show that the N-terminal region of APIP that is missing in the short isoform is required for activity. Together, these results confirm the involvement of APIP in the methionine salvage pathway, which plays a key role in many biological functions like cancer, apoptosis, microbial proliferation and inflammation.

## Introduction

**Figure 1:**
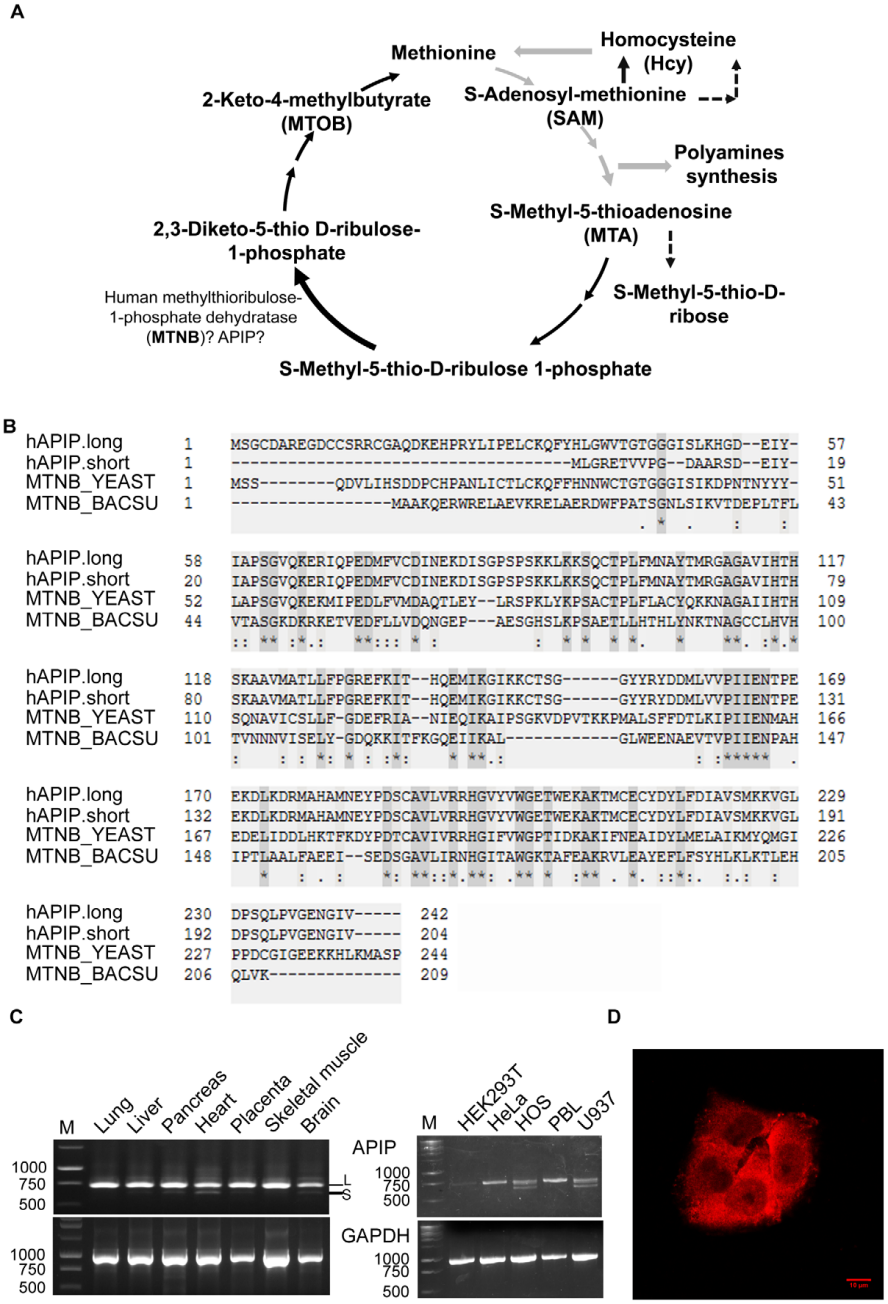
APIP as candidate to perform the step 2 of the methionine salvage pathway in human. (**A**) Schematic overview of the methionine salvage pathway. Black arrows: human specific routes; Black dashed arrows: *Shigella* specific routes; Grey arrows: common routes. (**B**) Sequence alignment of human APIP long and short isoforms (APIP.long and APIP.short) with mtnB enzymes from the yeast *Saccharomyces cerevisiae* and the bacteria *Bacillus subtilis*. (**C**) Analysis of the expression pattern of APIP mRNA by semi-quantitative RT–PCR. Left panel: Expression in tissues. This experiment was performed using a commercially obtained pre-normalized human multiple tissue cDNA panel (Clontech, panel I). Right panel: Expression in cell lines. The experiment was performed using total RNA preparations derived from human cell lines. The GAPDH gene was used as an internal control. For APIP at least two bands were detected (arrows) and confirmed to be the short and long isoforms after sequencing (APIP.long: 729 bp; APIP.short: 628 bp). M: Size marker; S: APIP.short; L: APIP.long. (**D**) Immunofluorence analysis of APIP into HeLa cells show a major cytoplasmic staining.

Methionine is an essential amino acid involved in major functions such as protein synthesis, formation of polyamines, DNA and protein methylation and protection against reactive oxygen species though the generation of glutathione [1]. In cells, the methionine that is not used for protein synthesis is converted into S-adenosylmethionine (SAM), the principal methyl donor (Figure 1A). Through the methylation cycle pathway, SAM can be converted back to methionine via the production of homocysteine (Hcy). SAM is also the precursor of polyamines such as spermine and spermidine. Polyamine synthesis leads to the production of 5′-methylthioadenosine (MTA) as a by-product [2]. The methionine salvage pathway allows cells to recycle the reduced sulfur in MTA back into methionine (Figure 1A) [3], [4]. The methionine salvage pathway and the polyamine synthesis seem to be tightly coupled, probably in order to maintain intracellular levels of SAM. For example, it has been shown that the level and activity of ornithine decarboxylase, the rate-controlling enzyme in polyamine synthesis, can be modulated by the first and last metabolites of the methionine salvage pathway: MTA and 4-methhylthio-2-oxo-butanoate (MTOB) [5]–[7]. The methionine salvage pathway may also have an important role in apoptotic processes as both MTA and MTOB were found to induce apoptosis [5], [8]. In eukaryotic cells, the methionine salvage pathway takes place in the cytosol and involves six enzymatic activities: MTA phosphorylase (MTAP, EC 2.4.2.28), 5′-methylthioribose-1-phosphate isomerase (mtnA, EC 5.3.1.23), 5′-methylthioribulose-1-phosphate dehydratase (mtnB, EC 4.2.1.109), 2,3-dioxomethiopentane-1-phosphate enolase/phosphatase (mtnC, EC 3.1.3.77), aci-reductone dioxygenase (mtnD, EC 1.13.11.54) and MTOB transaminase (EC 2.6.1.-) [4]. The transamination step can be catalyzed by a range of transaminases, which preferentially use aromatic and branched chain amino group donors [9].

The inventory of all enzymes involved in the methionine salvage has been recently achieved in yeast [9], but the pathway is still poorly functionally characterized in human. However, it has been attracting some interest for decades, mainly because its first enzyme, MTA phosphorylase, is frequently deficient in cancer cells and primary tumors [10]–[14].

At the beginning of our study, human MTAP was already functionally characterized as well as ADI1, that performs mtnD activity [15], [16]. Human mtnC enzyme was found to be ENOPH1 by homology with the *Bacillus subtilis* enzyme and its structure was solved by X-ray in the presence of a substrate analog to decipher its enzymatic mechanism [17]. There is strong evidence that MTOB transamination is mainly performed by glutamine transaminases [18]–[20]. The mtnA enzyme has been recently functionally characterized as MRI1, a protein that is induced in metastatic cells and promotes cell invasiveness [21].

Despite the conservation of the dehydratase step (mtnB, EC 4.2.1.109) in all organisms, it is one of the least studied enzymes in the pathway. Detailed enzymatic characterization was performed on recombinant *Bacillus subtilis* mtnB [22]. Subsequently, although homologs have been found in many organisms, functional characterization has been achieved so far only for *Pseudomonas aeruginosa*, *Saccharomyces cerevisiae* and *Tetrahymena thermophila* orthologs [23], [24]. The mtnB enzyme of *Tetrahymena thermophila* represents a divergent evolution as it was shown to be fused with mtnC and able to perform by itself three steps of the pathway (mtnB, mtnC, mtnD). Another case of gene fusion was observed in *Arabidopsis thaliana*, where the bifunctional enzyme encoded by At5g53850 probably mediates both mtnB and mtnC activities [22], [24]. The lack of detailed enzymatic studies for this enzyme may be due to the lack of commercial availability of the substrate and to the instability of the product [22].

The aim of this study was to complete the available information on the methionine salvage pathway in human cells by identifying the enzyme responsible for the dehydratase step (mtnB, EC 4.2.1.109). During the development of the HAMAP automatic annotation platform by the Swiss-Prot group [25] a sequence profile for the mtnB family was created in collaboration with the group of Prof. A. Danchin [24]. This profile as well as BLAST searches demonstrate that the closest human homolog of yeast MDE1/mtnB and *Bacillus subtilis* mtnB is a protein called APAF1-interacting-protein (HGNC gene symbol APIP, Q96GX9). We therefore changed its functional annotation in the UniProtKB/Swiss-Prot database into “Probable methylthioribulose-1-phosphate dehydratase (EC 4.2.1.109)”. One of the goals of our group being the experimental validation of bioinformatics predictions relevant to the function of human proteins, we decided to embark in the functional characterization of APIP. We used RNA interference in HeLa cells to validate the prediction that APIP is part of the methionine salvage pathway in human. Mutational analysis of the protein shows that mutation of the potential zinc binding site, inferred by the alignment of bacterial and yeast orthologs, abrogates APIP activity. We also demonstrate that an alternatively spliced isoform that lacks the first 38 N-terminal residues is catalytically inactive.

## Materials and Methods

### Sequence analysis

The putative human ortholog for the mtnB enzymes from *Bacillus subtilis* (O31668) and *Saccharomyces cerevisiae* (P47095) was searched by BLASTP analysis in the UniProtKB database version 15.14 [26]. Multiple sequence alignments were performed using Clustal Omega with the default parameters [27].

### Chemicals, antibodies, plasmids and cell culture

L-Methionine, Hcy, MTA, MTOB and SAM were purchased from Sigma. Anti-APIP and anti-V5 antibodies were from Santa-Cruz Biotechnology (D-20; 1/500 for Western blot) and AbD Serotec (MCA1360; 1/1000 for Western blot), respectively. Alpha-tubulin (α-tub) antibody was purchased from Sigma (T6074: 1/1000 for Western blot). ShRNAs were cloned into pRNAi-H1-Puro vector from Biosettia. APIP.long and APIP.short cDNAs were amplified by PCR from HeLa and HOS cells respectively using specific primers. Cloning into pcDNA3.2-V5 plasmid (Invitrogen) was achieved using the Gateway system (Invitrogen). V5APIP3HA, V5APIP3SA and V5APIP3SD were obtained though several rounds of site-directed mutagenesis using the Quickchange method (Stratagene). HeLa Kyoto cells (kind gift from Dr. Cécile Arrieumerlou) were cultured in complete or methionine free Dulbecco's modified Eagle's medium (DMEM, Invitrogen) supplemented with 10% fetal calf serum (Gibco, Invitrogen). Cells were incubated at 37°C in an atmosphere of 5% CO_2_ in air.

### Immunofluorescence

HeLa cells were fixed in 4% formaldehyde in PBS/pH 4 for 15 minutes at room temperature. After three washings of 5 minutes in PBS, cells were blocked in PBS supplemented with 5% donkey serum, 0.3% triton for 1 hour. APIP primary antibody (1/100) was incubated overnight in PBS containing 1% BSA, 0.3% Triton. After three washes in PBS, cells were incubated in Alexa donkey anti-goat secondary antibody (Invitrogen) for 1 hour.

### shRNA sequences, PCR and RT-PCR primers

sh1APIP: aaaaGAGCATCCAAGATACCTGATCttggatccaaGATCAGGTATCTTGGATGCTC

sh2APIP :

aaaaCTGCAAATGGTCACCCTGAATttggatccaaATTCAGGGTGACCATTTGCAG

Primers for APIP cDNAs amplification and RT-PCR:

APIP.long sense 5′- caccATGTCTGGCTGTGATGCTCG-3′,

APIP.short sense 5′-caccATGCTCGGGAGGGAGACTGTT-3′

APIP.long and APIP.short antisense 5′- CTTTCTTTTGGCTTAGACAATTCCATTTTC-3′

GAPDH sense 5′-TGAAGGTCGGTGTCAACGGATTTGGC-3′

GAPDH antisense 5′-CATGTAGGCCATGAGGTCCACCAC-3′

Primers for the generation of *Shigella metA* mutant:

MetA_3 and metA_4 were used for the amplification of a chloramphenicol cassette with flanking 60 bp sequences (lower case) that were homologous to *Shigella* chromosomal regions adjacent to the *metA* gene. MetA_1 and MetA_2 were used for screening of chloramphenicol resistant clones for *metA* replacement.

metA_1: 5′- GTGAGCGGCGAATACTA-3′

metA_2: 5′-TTCACTTGCTGAGGTGC-3′

metA_3: 5′-gttatcttcagctatctggatgtctaaacgtataagcgtatgtagtgaggtaatcaggttGTGTAGGCTGGAGCTGCTTCGA-3′

metA_4: 5′-gcacccgaaggtgcctgaggtaaggtgctgaatcgcttaacgatcgactatcacagaagaCATATGAATATCCTCCTTAGTTCC-3′

### Semi quantitative RT-PCR

A total of 1 µg of total RNA, isolated using the RNeasy kit (Qiagen), was used for reverse transcription with the Superscript II RNase H Reverse Transcriptase (Invitrogen) and random primers (Promega) according to manufacturer's protocol. The PCR amplification was performed with an annealing temperature of 56°C, one minute of elongation time and 26 cycles of amplification.

### Western blotting

HeLa cells were lysed with RIPA buffer for 30 minutes on ice and centrifuged for 20 min at 10 000 g to remove cellular debris. 80 µg of cells extract proteins were loaded on SDS-PAGE gel and transferred onto PVDF membrane.

### Transfection and cell growth assays

All the transfections were performed with Fugene HD (Roche) according to manufacturer's protocol. For transient experiments, HeLa cells were initially transfected with plasmids expressing shβGal, sh1APIP or sh2APIP. After 24 hours, cells were submitted to puromycin selection for 24 additional hours (2 µg/ml, Invitrogen). At 48 hours, cells were plated equally into 12 wells dishes (50 000 cells/well) and let to grow for 24 hours. For complementation experiments, cells were re-transfected with wild type or mutant V5APIP 72 hours after the initial transfection with shRNA plasmids. At 96 hours, media were switched to the different methionine, Hcy, MTA, MTOB, SAM media according to the experiments. Cells were counted at time point 144 hours (i.e. 48 hours after the media switch), using Alamarblue (Invitrogen) according to manufacturer's protocol. Alamarblue fluorescence was found to correlate very closely with manual cell counting (data not shown). To establish HeLa cell lines stably down-regulating APIP, HeLa cells transfected with shRNA vectors were selected with 1 µg/ml puromycin. Single colonies were isolated and analyzed for APIP down-regulation by Western blotting. The cell line with the best knock-down was harvested in nitrogen liquid. Cell growth assay in the different media was assessed at least three days after the cell lines were defrosted. For complementation in stable cell lines, equal amount of cells were plated in 12 well plates and transfected with 1 µg plasmid DNA/well. For co-expression experiments, the cells were transfected with 0.5 µg of each plasmid. The medium was changed 24 hours after transfection. Alamarblue measurements were performed after two days of incubation in the different media.

### *Shigella* infection experiment

Experiments were performed with *Shigella flexneri* serotype 2a strain 2457T with a mutation in *virG* (also called *icsA*) that prevent the bacteria move within the HeLa cell and spread from cell to cell [28]. This strain was a kind gift from Prof. Marcia Goldberg. The *Shigella virG* was transformed with the pNF106 vector that harbors a tetracyclin-inducible GFP construct (TETr-GFP) derived from the pZS21 vector [29]. The *metA* mutant was generated using an adapted method form from Datsenko and Wanner [30]. The chloramphenicol resistance cassette was amplified by PCR from a strain already carrying this cassette (see above for the primers). PCR products were transformed by electroporation into a 2457T virG strain that carries the pKM208 plasmid with the lambda recombinase system [31]. Chloramphenicol resistant clones were screened by PCR using primers located outside the *metA* gene (see above for the primers). Correct mutants were used as donors for transduction into *Shigella flexneri* serotype 2a strain 2457T *virG* using phage P1. The day before infection, HeLa cells were seeded in 96 wells plates (10^5^ HeLa cells/well). Two hours before infection, the medium (DMEM+FCS) was removed and replaced by meth^−^/MTA DMEM without FCS. Prior to cell infection, *Shigella* liquid cultures were grown at 37°C with shaking (200 rpm) to exponential phase (approximate optical density at 600 nm = 0.5). 1 ml of bacterial culture was spun down and re-suspended into 0.001% poly-L-Lysine (Sigma) in pre-warmed PBS and incubated for 15 minutes at 37°C. Treated *Shigella* were washed with PBS and re-suspended in pre-warmed meth^−^/MTA DMEM. Bacteria were added to cell monolayers at a multiplicity of infection (MOI) of 10 per HeLa cell, and the samples were centrifuged for 5 min at 600× *g*. After 30 min of incubation at 37°C, extracellular bacteria were killed by adding gentamycin (50 µg ml^−1^, Gibco) and GFP fluorescence was induced by adding anhydrotetracycline (*aTc, Sigma*). Infected cells were incubated at 37°C for up to 4 hours, trypsinized and fixed with 1% paraformaldehyde before flow cytometry analysis. The samples were analysed by a four-parameter (two scatter parameters, two fluorescence colours) flow cytometer equipped with a blue laser (488 nm) and an automated sample loader for 96 well plates (BD Bioscience HTS-LSR II SORP). HeLa cells and liberated individual *Shigella* were detected based on their particle size (forward scatter) and granularity (side scatter). The fluorescence properties of the gated particles were then analysed by exciting them with the blue laser and detection of the signals in the green (Ex488 LP502 PB530/30) and orange (Ex488 LP556 BP 585/42) channels. Data were stored in FCS 3.0 format and several descriptive statistics (mean, histogram) were calculated by the DIVA software © 2006, Becton, Dickinson and Company. The total number of *Shigella* per infected host cell (bacterial load) was calculated as follows: (arithmetic mean of total green fluorescence of infected HeLa cells - arithmetic mean of total green fluorescence of non-infected HeLa cells)/average green fluorescence of individual *Shigella*.

### Statistical analysis

Data are shown as mean ^+^/_−_ SD of results from at least three independent experiments. Statistical analysis was done by performing two-way ANOVA followed by Bonferroni post-test analysis (star marks in the figure legends: * = p<0.05, ** = p<0.01, *** = p<0.001).

## Results

### Identification of APIP as the candidate human mtnB enzyme

To confirm that APIP is the most likely human protein capable of performing mtnB activity, we performed a BLAST search on UniProtKB using the sequences of the *Bacillus subtilis* and *Saccharomyces cerevisiae* enzymes. APIP was found to be the best hit and presents 22.7% and 25.7% identity with the *Bacillus subtilis* and yeast enzymes, respectively. BLAST search using the human APIP sequence retrieves mtnB from both species, confirming that the three proteins are orthologs.

Two splice isoforms are described for APIP (APIP.long and APIP.short) [32]. APIP.short has 38 less residues in its N-terminus (Figure 1B).

We analyzed the expression pattern of the two APIP isoforms in different tissues and cell lines (Figure 1C). APIP.long was expressed in all the samples tested, presuming a ubiquitous expression. APIP.short was expressed at lower levels and detected in heart, brain, pancreas, liver and placenta. APIP.short was not detected in HeLa and PBL cells but was well amplified in HOS and U937 cells.

In accordance with its putative role in the methionine salvage pathway, APIP was detected mainly in the cytoplasm of HeLa cells by immunofluorescence (Figure 1D).

### Transient silencing of APIP in HeLa cells abrogates their growth in meth^−^/MTA medium

**Figure 2:**
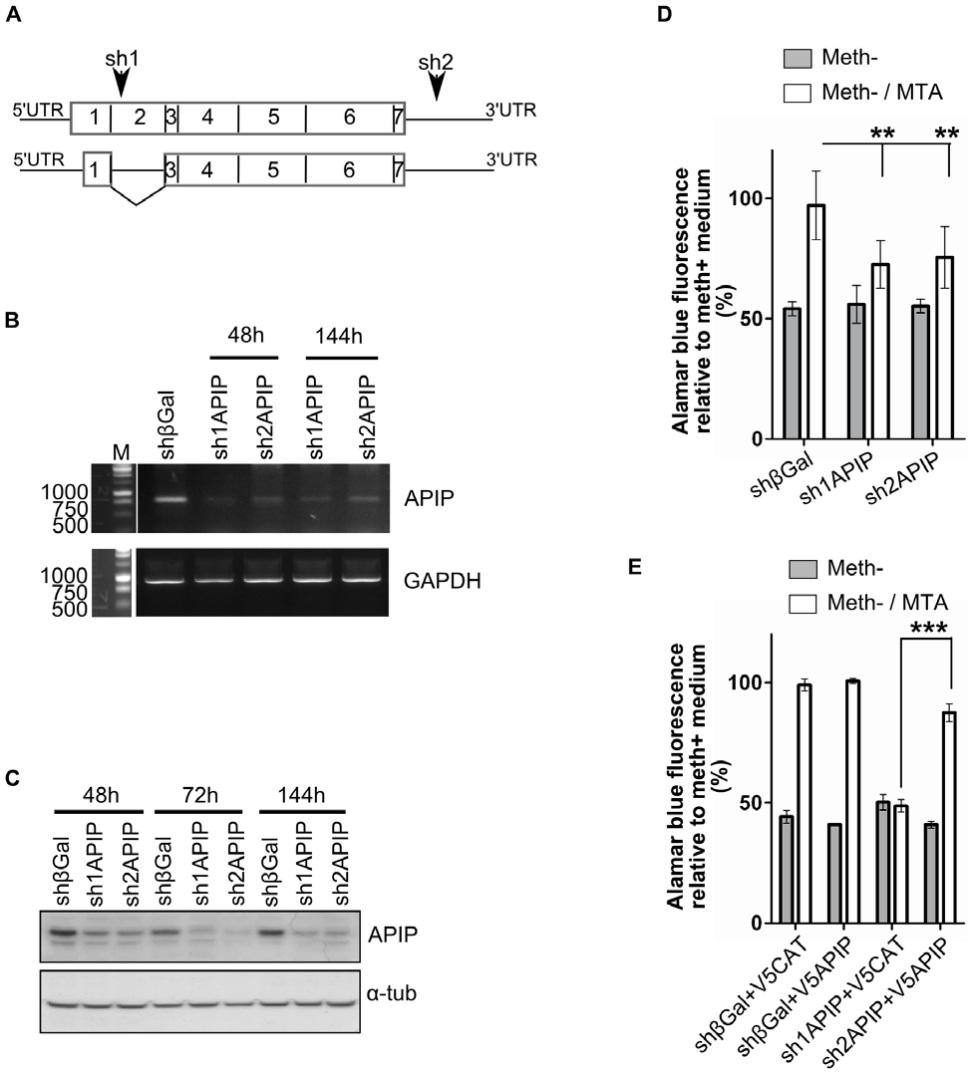
Transient silencing of APIP decreases the growth of the HeLa cells in MTA medium. (**A**) Schematic representation of the two mRNAs isoforms of APIP. Sequence positions of the shRNAs used in the study (sh1APIP and sh2APIP) are indicated by arrows. (**B**) Semi-quantitative RT-PCR analysis of APIP silencing 48 h and 144 h after transfection with plasmids expressing shRNAs. A plasmid expressing shRNA against β-galactosidase (shβGal) was used as a negative control. The GAPDH gene was used as an internal control. (**C**) Western blot analysis of APIP silencing 48, 72 and 144 hours after transfection with plasmids expressing shRNAs. All lanes were loaded with 80 µg of cell lysate proteins. Anti α-tubulin (α-tub) was used as a loading control. The bands present below APIP were nonspecifically stained with the anti-APIP antibody. (**D, E**) Cell growth analysis of HeLa cells transiently silenced for APIP. Alamarblue fluorescence is expressed relative to the fluorescence of HeLa cells transfected with the same plasmids and cultured in normal methionine media. (**D**) 96 hours after transfection with plasmids expressing shRNAs, the same number of cells was grown for 48 hours either in complete media, or in methionine free media complemented or not with MTA, and their end point growth was measured by Alamarblue fluorescence (**E**) 72 hours after transfection with plasmids expressing shRNAs, cells were transfected with plasmids expressing the N-terminally V5-tagged APIP protein (V5APIP) or the N-terminally V5-tagged chloramphenicol acetyltransferase protein (V5CAT). 24 hours later, the same number of cells was grown for 48 hours either in complete media or in methionine free media complemented or not with MTA, and their end point growth was measured by Alamarblue fluorescence.

Cells competent for the methionine salvage pathway, which recycles MTA to methionine, should be able to grow in meth^−^/MTA medium. To verify that HeLa cells have a functional methionine salvage pathway, we compared their proliferation in meth^+^, meth^−^ or meth^−^/MTA medium using Alamarblue fluorescence (Figure 2D). Cells treated with short hairpin RNA (shRNA) against the β-galactosidase (shβGal) for 96 hours (used as control cells) presented an average two-fold lower proliferation in meth^−^ medium than in meth^+^ medium. However, proliferation of cells in meth^−^/MTA was not significantly different than in meth^+^, confirming previous observations [33], and showing that HeLa cells' methionine salvage pathway is not affected by potential off-target effects due to shRNA treatment.

To confirm the involvement of APIP in the methionine salvage pathway, we transiently silenced APIP using shRNA in HeLa cells and looked if it affects their growth in meth^−^/MTA medium. For this purpose, we used two shRNA constructions: sh1APIP and sh2APIP, that target the cDNA region of APIP.long and the 3′UTR of the two isoforms, respectively (Figure 2A). RT-PCR analysis showed a significant knockdown of APIP mRNA 48 hours after transfection and until 144 hours for both sh1APIP and sh2APIP (Figure 2B). At protein level, both APIP shRNA constructs induced a slight depletion 48 h after transfection. The depletion was stronger at 72 hours and lasted until 144 hours (Figure 2C).

Silencing of APIP with either sh1APIP or sh2APIP reduces the proliferation of the HeLa cells in meth^−^/MTA to almost the same levels as in meth^−^, indicating that the methionine salvage pathway was impaired by APIP silencing (Figure 2D). To further confirm the specificity of the shRNA phenotype, we performed a rescue experiment by overexpressing N-terminally V5-tagged APIP.long (V5APIP) in shRNA treated cells. Overexpression of N-terminally V5-tagged chloramphenicol acetyltransferase (V5CAT) was used as control. Transfection with rescue plasmids was performed 72 h after transfection with shRNA plasmids and the proliferation of the cells in the different media was assessed as described above. As observed previously, proliferation of HeLa cells in meth^−^ was reduced two fold as compared to meth^+^. Overexpression of V5APIP or V5CAT in control (shβGal-treated) cells did not perturb their capacity to grow in meth^+^. However, overexpression of V5APIP, but not V5CAT, rescued the proliferation of the HeLa cells silenced for APIP with sh2APIP in meth^−^/MTA. Taken together, these results suggest that APIP is indeed involved in the methionine salvage pathway.

### Stable knockdown of APIP in HeLa cells specifically affects growth in MTA and SAM media and depletes intracellular levels of methionine

**Figure 3:**
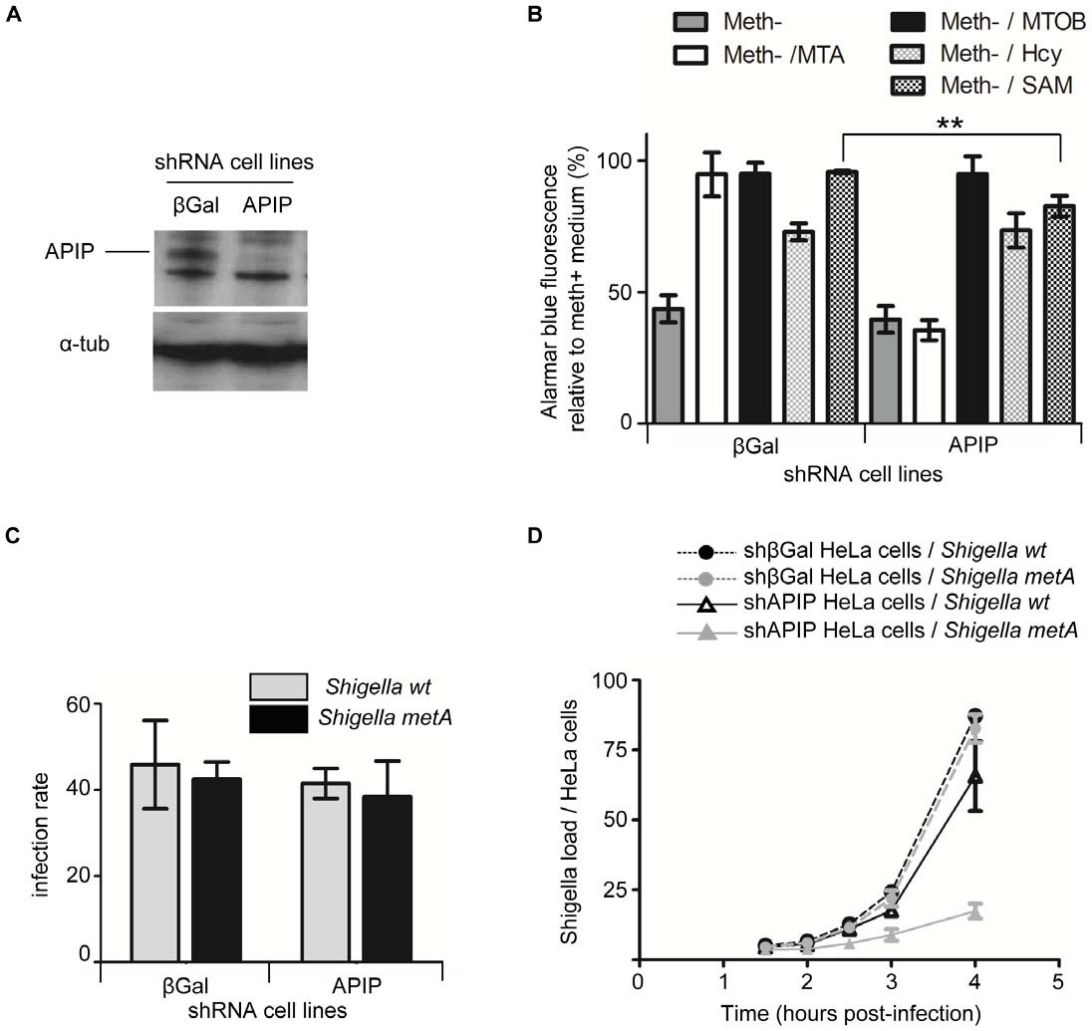
Stable knockdown of APIP specifically affects growth in MTA and depletes intracellular levels of methionine. (**A**) Western blot analysis of APIP stable knockdown HeLa cells. (**B**) Alamarblue cell growth analysis of control (shβGal) and APIP knockdown stable cell lines in MTA, MTOB, Hcy and SAM media. Results are expressed relative to the growth of the same cell lines in methionine medium. (**C, D**) Effects of APIP depletion on infection by wt *Shigella* and by a *Shigella* strain auxotrophic for methionine (*Shigella metA*). HeLa cells were switched to MTA for two hours and then infected in the same media with wt and mutant form of *Shigella flexneri* serotype 2a strain 2457T for 4 hours. Parameters of infection were monitored by flow cytometry as described in material and methods. (**C**) Measurement of infection rates (i.e percentage of infected HeLa cells) in each condition. No significant difference was observed. (**D**) Time course analysis of the *Shigella* load shows that the growth of *Shigella metA* is severely diminished in HeLa cells silenced for APIP in the absence of methionine in the cell culture medium.

To characterize further the involvement of APIP in the methionine salvage, we engineered HeLa cells stably silenced for APIP. The knockdown was confirmed by Western blot (Figure 3A). We then studied the capacity of these HeLa cells to grow after two days incubation in media containing different sources of methionine (Figure 3A). As expected, both control cells and APIP knockdown cells presented a decreased proliferation of around 2 fold in meth^−^ medium. As observed during transient experiment, knockdown of APIP resulted in a reduced cell proliferation in meth^−^/MTA, equivalent as the one observed in meth^−^. As expected, knockdown of APIP did not affect proliferation in meth^−^/MTOB medium, showing that APIP is involved in the methionine salvage pathway downstream of MTA but upstream of MTOB generation.

SAM is a precursor for both the methylation cycle and the methionine salvage pathway (Figure 1A). Knockdown of APIP slightly but significantly reduced the proliferation of HeLa cells in meth^−^/SAM medium, to a rate of 90% relative to control HeLa cells. Based on this result we can assume that in this condition most of the methionine is recycled by the methylation pathway. The last step of the methylation cycle uses homocysteine as a substrate for methionine synthase. Although HeLa cell growth was slightly decreased in meth^−^/Hcy medium as compared to methionine medium as previously described [34], [35], control and APIP knockdown HeLa cells proliferate similarly in meth^−^/Hcy medium. Taken together, these results suggest that down regulation of APIP acts specifically on the methionine salvage pathway, without affecting the methylation pathway.

To confirm that intracellular methionine levels were indeed affected by APIP knockdown, we performed HeLa cells infection experiments with either a wild type (wt) strain or a methionine auxotroph mutant (*Shigella metA*) of *Shigella flexneri*. *Shigella* is able to synthesize methionine from pyruvate via its own biosynthetic pathway and is also able to recycle methionine through the methylation cycle pathway [24], [36]. However, *Shigella* (Figure 1A) does not possess a functional methionine salvage pathway and is therefore not able to use MTA as a source a methionine. While not all the transporters capable of importing methionine in enterobacteria such as *Shigella* have been fully characterized at the molecular level, it is known that *Shigella* is able to use the methionine from the host [37]–[41]. The methionine auxotroph mutant *Shigella metA* was obtained by deletion of the homoserine O-acetyltransferase enzyme (EC 2.3.1.31, *metA*) from the biosynthetic pathway. We first looked at the percentage of HeLa cells infected by both bacterial strains in MTA medium (Figure 3C). No significant difference was observed between the two strains, meaning that the deletion of *metA* did not affect the invasion capability of *Shigella*. The percentage of infected HeLa cells was also not significantly changed by the silencing of APIP excluding an involvement of APIP in the entry process of *Shigella* into the cells. Next, we looked at *Shigella* growth inside HeLa cells by measuring the load of *Shigella* per HeLa cell over time (Figure 3D). Wt and *Shigella metA* grew similarly in control shβGal HeLa cells. In these cells, *Shigella metA* probably compensated their deficiency in methionine biosynthesis by recruiting the host methionine provided by the methionine salvage pathway from MTA. In APIP knockdown cells, the growth of wt *Shigella* was not significantly affected, but the growth of *Shigella metA* was dramatically inhibited, suggesting insufficient availability of methionine inside the host cells as a consequence of disruption of the methionine salvage pathway.

### Mutational analysis of APIP activity in the methionine salvage pathway

**Figure 4:**
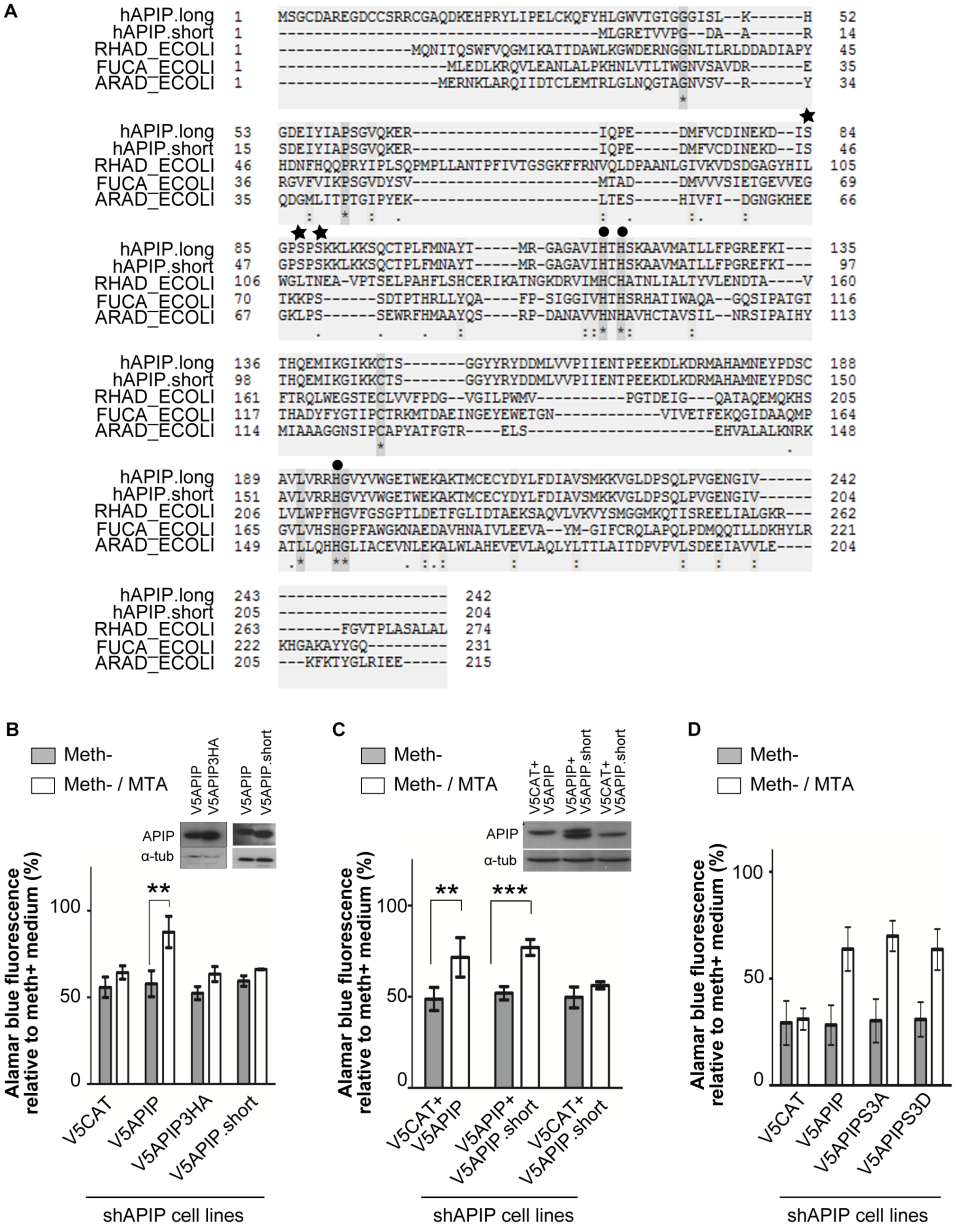
Mutational analysis of APIP activity. (**A**) Sequence alignment of APIP.long with bacterial enzymes of the same family (class II aldolases). RHAD: Rhamnulose-1-phosphate aldolase (P32169, EC 4.1.2.19), ARAD: L-ribulose-5-phosphate 4-epimerase (P08203, EC 5.1.3.4), FUCA: L-fuculose-1-phosphate aldolase (P0AB87, EC 4.1.2.17). (**B, C, D**) Alamarblue cell growth analysis of APIP knockdown HeLa cells rescued with mutant forms of V5APIP in MTA medium. Results are expressed relative to the growth of the HeLa cells treated the same way in methionine medium (**B**) V5APIP mutated for the potential zinc binding site (V5APIP3HA) and V5APIP.short are not able to restore cell growth in MTA medium. Expression of V5APIP, V5APIP3HA and V5APIP.short was controlled by Western blot in APIP stable knockdown HeLa cells. (**C**) Co-expression of V5APIP.short did not affect the growth rescue in MTA conferred by V5APIP. V5CAT was used as control so that the amount of plasmid DNA used for transfection was constant in the three conditions. (**D**) No difference was observed in the rescue efficiency of V5APIP and V5APIP mutated at potential phosphorylation sites (V5APIP3SA and V5APIP3SD).

mtnB is part of the divalent metal ion-dependent aldolase class II family which includes bacterial L-ribulose-5-phosphate 4-epimerase (araD), L-fuculose phosphate aldolase (fucA) and rhamnulose-1-phosphate aldolase (rhaD) (Figure 4A). The X-ray structures of *E.coli* AraD, FucA and RhaD are solved and have helped to decipher their molecular mechanism [42]–[47]. These aldolases use zinc as a co-factor. As expected, the three histidines involved in zinc binding are conserved in APIP. By site-directed mutagenesis, we changed these histidines into alanines and tested the effect of these mutations on APIP activity in the methionine salvage pathway. As shown in Figure 4B, overexpression of APIP triple-alanine mutant (V5APIP3HA) failed to rescue the growth defect of APIP knockdown HeLa cells in meth^−^/MTA media, although correct expression of V5APIP3HA was checked by Western blot (Figure 4B, panel). Therefore, as expected by homology to other metal ion-dependent aldolase class II enzymes, these three histidine residues are important for APIP activity. We next assessed the activity of the APIP.short isoform by overexpressing it in APIP knockdown HeLa cells. As shown in Figure 4B, although expressed at a similar level as V5APIP, V5APIP.short failed to rescue the growth defect in MTA medium. Despite the strong similarity between the two isoforms, the absence of the first 38 amino acids disrupts APIP activity in the methionine salvage pathway. We then investigated if the inactive APIP.short could compete with APIP for activity. Overexpression of V5APIP.short did not affect the capacity of control shβGal HeLa cells to grow in meth^−^/MTA medium (data not shown). This observation suggests that V5APIP.short does not affect the activity of endogenous APIP. To confirm that V5APIP.short does not act as an inhibitor of full length APIP, we co-expressed both V5APIP and V5APIP.short in the APIP knockdown stable cell line. A 1∶1 ratio of DNA plasmids was used during transfection. Controls were obtained by co-expressing V5CAT with either V5APIP or V5APIP.short at the same ratio. As observed in Figure 4C, co-expression of V5APIP.short did not affect the growth rescue by V5APIP in meth^−^/MTA medium. From these results, we conclude that the short isoform of APIP does not act as a negative regulator of the full length isoform.

APIP was found to be phosphorylated at Ser-87 and Ser-89 in large scale phosphoproteome studies performed on HeLa S3 cells [48] and human embryonic stem cells HUES 9 [49]. Another serine located just upstream (Ser-84) might also be phosphorylated (Prof. C. Arrieumerlou, personal communication). We mutated these three serines either in alanine (V5APIP3SA), in order to abrogate potential phosphorylation, or in aspartic acid to mimic phosphorylation (V5APIP3SD). As shown in Figure 4D, both V5APIP3SA and V5APIP3SD were able to rescue APIP knockdown phenotype in meth^−^/MTA medium similarly as the wt form of APIP (V5APIP). Hence, phosphorylation at these sites seems not to be required for APIP activity.

## Discussion

Despite its discovery many years ago [3], [50], the methionine salvage pathway is still incompletely described in terms of enzymatic composition, especially in human. In this study, we functionally characterized APIP as the human ortholog of mtnB, the enzyme catalyzing the dehydratase step. While we were writing this manuscript, a paper came out presenting similar results and confirming the function of APIP into the methionine salvage pathway [51]. MtnB belongs to the divalent metal ion-dependent aldolase class II family [42]–[47] that comprises mainly bacterial enzymes. The characteristic features of this family are conserved in APIP. The three histidines expected to be responsible for metal binding based on homology with mtnB [22] are present in APIP and essential for activity. APIP also possesses a conserved glutamate that was shown to be important for the deprotonation of the substrate and residues that were shown to be required for phosphate binding [45]. *Bacillus subtilis* mtnB as well as related enzymes RhaD, AraD and FucA were shown to form homotetramers [22], [45]–[47]. The capacity of human APIP to oligomerize should be investigated further.

APIP.short, which lacks the 38 N-terminal residues, is not able to replace the long isoform in the methionine salvage pathway. Some of these N-terminal residues are well conserved across mtnB eukaryotic orthologs and may be key for enzymatic activity. We also show that APIP.short does not act as an inhibitor of the full length isoform, suggesting that it does not bind to APIP nor compete for the substrate. Interestingly, we were not able to find this short isoform in ESTs or full length mRNA collections in organisms other than human.

Both APIP isoforms were previously shown to interact with APAF-1, thereby competing for binding to caspase-9 [52]. Accordingly, APIP overexpression inhibited mitochondrial apoptosis induced by drugs like etoposide and staurosporine whereas APIP down-regulation increased susceptibility to apoptosis in mouse skeletal muscle cells C2C12. Moreover, the same study showed that APIP is induced in mice muscles upon ischemia and hypoxia, and that its overexpression suppresses ischemia/hypoxia-induced death. The effects of APIP during ischemia/hypoxia were first correlated with its APAF-1-related anti-apoptotic activity, but it was later demonstrated they could occur independently of the presence of APAF-1, via activation of AKT and extracellular signal-related kinases MAPK3 (ERK1) and MAPK1 (ERK2) [32]. Although we did not find any effect of APIP up and down-regulation on apoptosis in HeLa cells (data not shown), the modulation of apoptosis by APIP has been recently confirmed in lymphoblastoid and HEK293 cells [51]. Moreover, APIP was also shown to regulate caspase-1-related inflammatory responses (pyroptosis) in the same cells. Because of the central role of methionine and SAM, disruption of the methionine salvage pathway may affect the cells in many manners. Accordingly, the same study showed that MTA itself could regulate pyroptosis and that APIP acts on pyroptosis via the methionine salvage pathway.

Previous studies have shown that many cancer cell lines were not able to grow in meth^−^/Hcy media [34]. This phenomenon was named methionine dependency [53] and led to the development of new therapeutic strategies, such as methionine restriction diet [54]–[58]. This observation has brought interest into the methionine salvage pathway, firstly, because MTAP is deficient in many cancer cell lines and secondly, because four methionine-dependent cell lines could be rescued by the addition of MTOB in the media [35]. However, it was shown that, despite the strong correlation between methionine dependency and the loss of MTAP expression, over-expression of MTAP in deficient cell lines rescued growth in meth^−^/MTA but not in meth^−^/Hcy [35]. In our study we show that APIP knockdown does not perturb the growth of the HeLa cells in meth^−^/Hcy. Taken together, these two observations indicate that deficiency in methionine salvage pathway is not responsible for cell methionine dependency.

Finally, during the last 5 years, genetic studies revealed potential links between APIP expression levels and diseases. For example, APIP was found to be amplified and up-regulated in squamous carcinoma cells lines from tongue and larynx [59]. Inversely, APIP was found to be down-regulated at mRNA and protein levels in non-small cell lung carcinoma cells and tumors [60]. Genetic variants located in the 3′ UTR region of APIP were found to be associated with lung disease severity in cystic fibrosis [61]. Finally, using a genome wide association study, a common SNP (rs514182) associated with reduced expression of APIP has been shown to be linked to increased susceptibility to both pyroptosis caused by *Salmonella* infection and to the chemotherapeutic agent carboplatin [51]. Interestingly, this mutation was also linked to improved survival of individuals with systemic inflammatory response syndrome. Possible deregulation of methionine metabolism in those diseases should be investigated further.
